# Repair of the Perineum, Using Silk-Worm Gut and Leaving the Sutures in for Two or Three Months

**Published:** 1913-10

**Authors:** Gaston Torrance

**Affiliations:** Birmingham, Ala.


### REPAIR OF THE PERINEUM, USING SILK-WORM GUT AND LEAVING THE SUTURES IN FOR TWO OR THREE MONTHS.

By Gaston Torrance, M. D., Birmingham, Ala.

About a year ago I was asked to repair a perineum that was badly lacerated following a forceps delivery: Silk worm gut sutures were used and tied snugly, giving good coaptation; three of four sutures were used on either side and were knotted together in two bundles.

At the end of two weeks the attending physician removed the sutures on one side but evidently overlooked those on the opposite side. About two or three months later the patient came to me to have these removed. The result on this side was better; there was no irritation around the sutures and they were removed without pain.

Since this time I have used this method on over forty cases of repair and am sure that my results are decidedly improved.

I recently removed the sutures from a patient who came from her home town five months after operation with almost a perfect result.

